# A Novel Method to Rank Influential Nodes in Complex Networks Based on Tsallis Entropy

**DOI:** 10.3390/e22080848

**Published:** 2020-07-31

**Authors:** Xuegong Chen, Jie Zhou, Zhifang Liao, Shengzong Liu, Yan Zhang

**Affiliations:** 1 School of Computer Science and Engineering, Central South University, Changsha 470075, China; csucxg@csu.edu.cn (X.C.); 174712114@csu.edu.cn (J.Z.); 2 School of Information Technology and Management, Hunan University of Finance and Economics, Changsha 410205, China; 3 School of Engineering and Built Environment, Glasgow Caledonian University, Glasgow G4 0BA, UK; yan.zhang@gcu.ac.uk

**Keywords:** influential nodes, Tsallis entropy, SIR model

## Abstract

With the rapid development of social networks, it has become extremely important to evaluate the propagation capabilities of the nodes in a network. Related research has wide applications, such as in network monitoring and rumor control. However, the current research on the propagation ability of network nodes is mostly based on the analysis of the degree of nodes. The method is simple, but the effectiveness needs to be improved. Based on this problem, this paper proposes a method that is based on Tsallis entropy to detect the propagation ability of network nodes. This method comprehensively considers the relationship between a node’s Tsallis entropy and its neighbors, employs the Tsallis entropy method to construct the TsallisRank algorithm, and uses the SIR (Susceptible, Infectious, Recovered) model for verifying the correctness of the algorithm. The experimental results show that, in a real network, this method can effectively and accurately evaluate the propagation ability of network nodes.

## 1. Introduction

With the expansion of the Internet, people are paying increasingly more attention to social networks (WeChat, Facebook, and Instagram). When analyzing social networks, it becomes more important to mine influential nodes. For example, the collaborator network [1] analyzes academic research to distinguish the different academic influences of different authors, thus providing researchers with scientific evidence, especially those who are not familiar with a certain field, so that they can quickly enter the field. Furthermore, it plays an important supporting role in promoting the exchange of science and technology. Sentiment analysis or opinion mining [2] uses natural language processing tools in order to extract subjective information from text to assess the attitudes of some users, provide enterprises with product promotion channels, understand user psychology, and obtain market information, which has important reference significance. Online advertising [3] can select the most influential users (online celebrities) to specifically show the users with brand affinity. It can be used for product recommendation, and it can use the celebrity effect to continuously expose consumers to products, which is of great significance for product marketing. In research, influential nodes are considered to have better communication capabilities, which mean that they can disseminate information to more network users; therefore, identifying influential nodes is an important factor in the successful dissemination of information in social networks.

In the research of node influence in complex networks, the earliest method is based on the degree of nodes, such as the degree centrality that is based on centrality [4], and they all use the network locations of nodes to evaluate the node influence. These methods mainly evaluate the importance of nodes based on the number and relative distribution of connected edges, which is simple and effective; however, the degree of nodes is based on the local information method and, thus, the influence and function of nodes in the whole network are not effectively described. Furthermore, the importance of complex network nodes also depends on the network structure around them. In the study of the structural complexity of complex networks, some scholars have made many related researches on the structural characteristics of networks, such as the closeness centrality [5], betweenness centrality [6], eigenvector centrality [7], Katz centrality [8], and entropy. Entropy is an important method for evaluating the characteristics of the network structure. When entropy is used to evaluate a complex network, the more orderly the structure of the complex network is, the smaller the entropy value, and vice versa. At the same time, entropy can also be used to describe the complexity of the overall network structure and the statistical characteristics of a complex network.

For example, in 2012, Chen et al. [9] proposed the structural entropy to measure the structural characteristics of complex networks. Xu et al. [10] proposed a path entropy-based approach to link predictions in real networks. Qiao et al. [11] proposed a new mechanism for quantitatively measuring centrality based on a graph decomposition and domain node entropy redefining entropy centrality model. However, these traditional methods lack the ability to capture the global information of nodes, and they seldom consider the locations of nodes in the network. To solve these problems, this paper proposes a novel method for evaluating the propagation ability of the nodes in a network: TsallisRank method (TRank). This method combines a node’s propagation ability and the degree of a node’s neighbors, fully measures the correlation between the primary and secondary neighbors of a node, and uses the Tsallis entropy in order to evaluate the complexity of the network structure.

The rest of this paper is organized, as follows. The Section 2 outlines the related work that influences this study, and the Section 3 introduces the motivation and details of the method in detail. The Section 4 gives the details of the experimental results and evaluation results, and the experimental conclusions are written in the Section 5.

## 2. Related Works 

So far, many scholars have put forward many measures and methods in the research of the influence of complex networks [12,13]. Among them, there are influence analysis methods based on a node’s own attributes, mainly including the evaluation method that is based on the degree of nodes, the K-shell decomposition method, and so on. The degree centrality analysis method was proposed by Bonacich P [14]. It mainly considers the size of the degree of a node. The larger the degree is, the greater its influence. Kitsak et al. [15] proposed a fast node ranking method, called K-shell decomposition, which considers the network locations of nodes when determining the influence ability. Bae & Kim et al. [16] considered the degree of nodes and the core of their influence, which is also more concise. Zeng et al. [17] proposed a new method that is based on K-shell decomposition and mixed degree decomposition (MDD), in which MDD weighed the remaining degree and the reduced degree of nodes after K-shell decomposition. On the basis of K-shell, Wang et al. [18] considered not only the K value after node decomposition, but also the number of iterations each time.

Another method is the ranking method based on the centrality of eigenvectors, which considers the quantity and quality of adjacent nodes at the same time. The main method makes some improvements that are based on the PageRank and hits algorithm. PageRank algorithm defines the influence propagation of nodes as the important score propagation. In the initial state of iteration, each node in the network distributes its own PageRank value equally for the nodes to which it points, update the PR value of each node until the algorithm converges, and finally determine the importance of the nodes according to the final PR value. Weng, Lim, et al. [19] proposed a twitter rank algorithm based on PageRank, which is used to measure the topic similarity between users and the impact of the link structure. Chen et al. [20] analyzed the three aspects of post quality, the proportion of forwarding behavior, and interest similarity; calculated the relative impact of forwarding behavior; and, improved PageRank with the unique structural and behavioral characteristics of a microblog network. Wang et al. [21] proposed a consistency algorithm called ConformRank to find the most influential users. Emotion integration refers to how users maintain the same emotion as the original users. The consistency weight evaluates the consistency of user emotion.

In addition, entropy is an effective tool to describe the complexity and uncertainty of the social impact, and so it has been widely used in social networks. Peng [22] proposed two concepts, the friend entropy and the interaction frequency entropy, in order to measure the social impact. Sathanur and Jandhyala et al. [23] introduced the transfer entropy to measure the impact of directed causality. Yin L and Deng y [24] used heuristic rules to measure the utility of each neighbor in the network and the Shannon entropy to measure the uncertainty of each node. Xiao et al. [25] proposed a new structural entropy based on the automorphism partition to accurately quantify the heterogeneity or disorder of a network system. Nie t et al. [26] considered the local information of the correlation between each node and its neighboring nodes to propose the mapping entropy.

## 3. Motivation and Proposed Approach

This section mainly explains the origin and algorithm flow of the Tsallis entropy algorithm, in which the final algorithm and its flow is derived step by step.

### 3.1. Tsallis Entropy

Entropy is a concept in physics. Entropy connects a microstate with a macro characteristic and uncertainty with information measurement, and it measures order and disorder. In 1988, the Brazilian physicist Tsallis [27] proposed the Tsallis entropy that is based on the existing Boltzmann entropy. Its formula is as follows.

(1) $$S_{q}=k\frac{1-\sum_{i=1}^{W}P_{i}^{q}}{q-1}\left(q\epsilon R\right)$$

where $S_{q}$ is the value of the Tsallis entropy, W is the number of particles in a micro system, k is the Boltzmann constant, q is the Tsallis parameter that describes the interaction between elements, and $p_{i}$ represents the probability of occurrence of microparticles. In this paper, the Tsallis entropy is used for detecting the propagation ability of complex network nodes based on the Tsallis entropy formula in order to measure the complexity of a network structure. The formula is as follows.

(2) $$T_{i}=\sum_{j=1}^{W}\frac{p_{ij}{}^{q_{ij}}-p_{ij}}{1-q_{ij}}$$

where $T_{i}$ represents the entropy value of node i in the local area network; and node i and the nodes directly connected to this node constitute a network with a radius of 1, which is called the local area network of node i. $p_{ij}$ represents the probability set of neighbor j around node i in the local area network, W is the number of nodes in the local area network, and $q_{ij}$ represents the system parameters of a neighbor j of node i. When calculating the propagation ability of a complex network, this paper uses the closeness centrality to represent the interaction parameters of nodes and the system parameters, which can improve the overall effect of nodes in the network. It is reasonable to evaluate the structural complexity of complex networks.

### 3.2. TsallisRank

**Figure 1:**
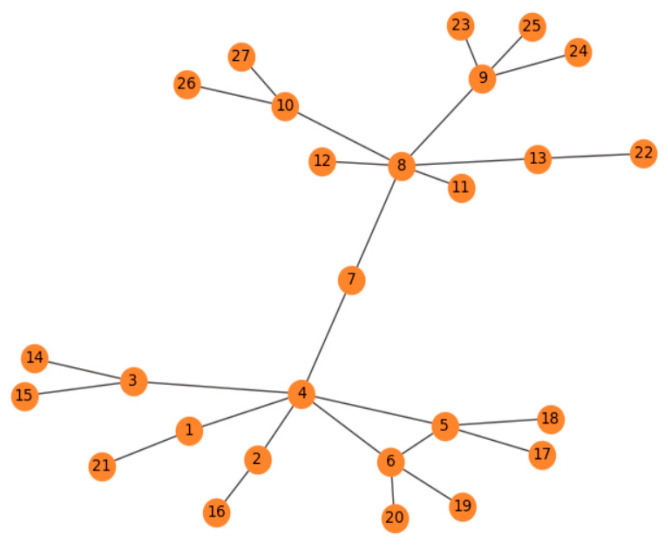
Node network diagram.

In the research of influence in complex networks, many methods are based on the degree of nodes. However, only depending on the degree cannot fully measure the influence of a node. If the degree of a node’s neighbors can be considered, it may improve the accuracy of the influence of the whole node. For example, node 4 and node 8 have the same degree centrality of 6, and they will have the same propagation ability, as shown in Figure 1. However, the two neighbors of node 8 are node 11 and node 12, both of which have no neighbors; therefore, the propagation ability of node 8 should be smaller than that of node 4 and, thus, the propagation ability of node 8 will be different. Therefore, we think that the propagation ability of a node is positively related to its neighboring nodes’ degree, and so we propose the TsallisRank algorithm.

**Figure 2:**
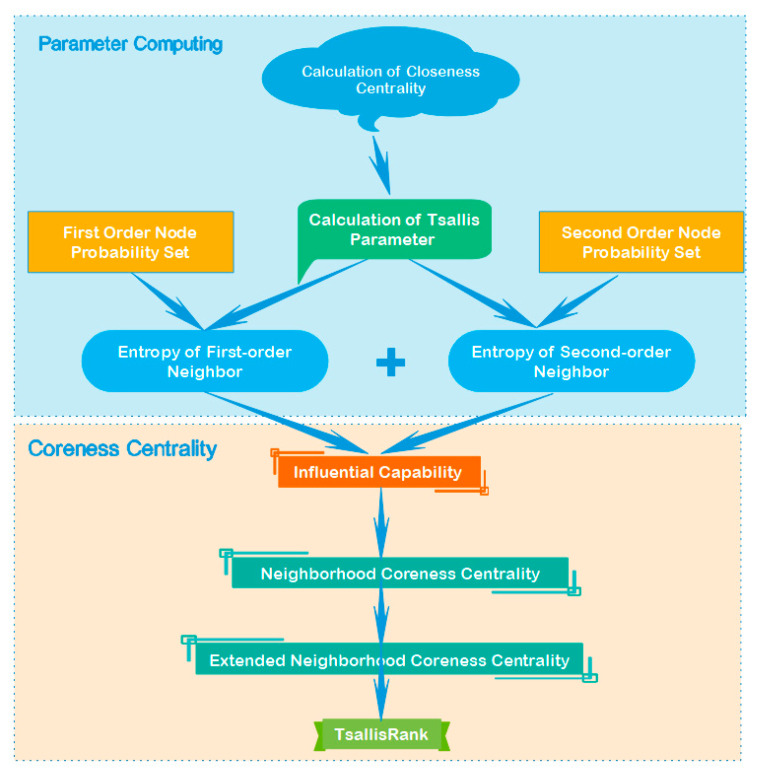
Flow chart of the TsallisRank algorithm.

The TsallisRank algorithm that is based on the Tsallis entropy is mainly divided into two parts. The first part is the calculation of all kinds of parameters to prepare the following formula. First of all, calculate the compactness centrality for each node, and then use the compactness centrality to calculate the Tsallis parameter q, and then build a local area network. Each node calculates the first-order neighbor and second-order neighbor probability set. Finally, the Tsallis parameters and probability sets are used to calculate the first-order neighbor entropy and the second-order neighbor entropy, respectively. In the second part, the purpose is to integrate the two kinds of neighbor entropies, calculate the propagation ability, and then calculate the final TsallisRank through the two neighborhood cores. Please refer to Figure 2 for the specific steps.

#### 3.2.1. Parameter Computing

- Calculate compactness centrality

For a network, we define G=(V,E) as the connected graph, n=|V| as the number of nodes, m=|E| as the number of edges, d(i,j) as the shortest path between node i and node j, and $C_{i}$ as the tight centrality of node i. It is defined, as follows.

(3) $$C_{i}=\frac{n-1}{\sum_{j\neq i}d\left(i,j\right)}$$

- Calculate the Tsallis parameters

Kitsak et al. [15] believe that the influence ability of a node is determined by its network location. Therefore, the most influential nodes will maintain closer relationships with their surrounding nodes. $q_{i}$ represents the Tsallis parameter of node i, and $q_{i}$ is defined, as follows, where $C_{max}$ is the maximum value of the tight centrality in the network.

(4) $$q_{i}=1+C_{max}-C_{i}$$

- Calculating the probability set

First of all, we need to build a node local area network, which is called node i’s local area network. The degree of node i is represented by $k_{i}$, $N_{i}$ is the set of its neighbors, $k_{i}^{1}$ is the sum of the degrees of all the neighbors of node $v_{i}$, and $k_{i}^{1}=\underset{v_{j}\in N_{i}}{\sum }k_{j}$. Subsequently, $k_{i}^{2}$ is the sum of the degrees of the neighbors of node $v_{i}$, which is called the second-class neighbor in this paper, and $k_{i}^{2}=\underset{v_{j}\in N_{i}}{\sum }k_{j}^{1}$.

(5) $$p_{i}^{1}=\frac{k_{j}}{k_{i}^{1}}$$

(6) $$p_{i}^{2}=\frac{k_{j}^{1}}{k_{i}^{2}}$$

where $p_{i}^{1}$ is defined as the first-order probability set of node $v_{i}$, and $p_{i}^{2}$ is the second-order probability set of node $v_{i}$.

- Neighbor entropy

According to the inference of Equation (3), this paper uses the Tsallis value and probability set obtained above to replace Equation (3), and then formula 5 and formula 6 are obtained.

(7) $$Ts_{1}\left(v_{i}\right)=-\underset{v_{j}\in N_{i}}{\sum }\frac{{\left(p_{j}^{1}\right)}^{q_{i}}-\left(p_{j}^{1}\right)}{1-q_{i}}$$

(8) $$Ts_{2}\left(v_{i}\right)=-\underset{v_{j}\in N_{i}}{\sum }\frac{{\left(p_{j}^{2}\right)}^{q_{i}}-\left(p_{j}^{2}\right)}{1-q_{i}}$$

where $Ts_{1}\left(v_{i}\right)$ is the first-order neighbor entropy of node $v_{i}$ and $Ts_{2}\left(v_{i}\right)$ is the second-order neighbor entropy of node $v_{i}$.

#### 3.2.2. Coreness Centrality

- Ability to calculate the impact

The coefficient $\alpha_{i}$ is defined in this paper in order to integrate the first-order neighbor entropy and the second-order neighbor entropy. It is a ratio that combines the two entropy values organically.

(9) $$IC\left(v_{i}\right)=Ts_{1}\left(v_{i}\right)+\alpha_{i}Ts_{2}\left(v_{i}\right)$$

(10) $$\alpha_{i}=\frac{k_{i}^{2}}{max_{v_{h}\in V}\left(k_{h}^{2}\right)}$$

where $IC\left(v_{i}\right)$ represents the influence ability of node $v_{i}$, which describes the mutual influence ability of the primary and secondary neighbors of node $v_{i}$; and, $max_{v_{h}\in V}\left(k_{h}^{2}\right)$ represents the maximum value of the sum of the degrees of the secondary neighbors of a node in a network. The value field of $\alpha_{i}$ is $0<\alpha_{i}<1$.

- Computing the neighborhood core

Bae and Kim [16] put forward the concept of the neighborhood kernel in this paper when improving the K-shell algorithm. This paper will draw on this concept and it uses the following equation.

(11) $$Cnc\left(v_{i}\right)=\underset{v_{j}\in N_{i}}{\sum }IC\left(v_{j}\right)$$

The meaning of Cnc is that for node $v_{i}$, the IC value of all its neighbors can be summed to get the neighborhood core Cnc (core neighborhood centrality) of node $v_{i}$.

- TsallisRank

(12) $$TRank\left(v_{i}\right)=Cnc^{+}\left(v_{i}\right)=\underset{v_{j}\in N_{i}}{\sum }Cnc\left(v_{j}\right)$$

where *TRank* is the abbreviation of TsallisRank, which will be used in place of TsallisRank. For node $v_{i}$, by summing the Cnc of all its neighbors, we can get the extended core neighborhood of node $v_{i}$. In this paper, we set the TRank equal to $Cnc^{+}$, and finally we get the TRank.

### 3.3. Algorithm Description

According to the above formula explanation, in order to further understand the TRank algorithm, this paper gives the pseudo code as shown in Algorithm 1.
**Algorithm 1:** TRank algorithm.**Input**: Network G(V,E) Output: TRank Value for each node 1. Find neighboring nodes $N_{i}$ of node $v_{i}$ 2. Compute $q_{i}$ for node $v_{i}$ 3. **For** node $v_{j}$ in $N_{i}$ do 4. compute ratio1 = degree ($v_{j}$)/sum(degree(all neighbors of $v_{j}$)) 5.$\;Ts_{1}$ = (pow(ratio1,$\;q_{i}$) − ratio1)/(1-$q_{i}$) 6. **End For** 7. **For** node $v_{j}$ in $N_{i}$ do 8. compute second_neighbor_degree= the degree of the second neighbor for node $v_{j}$ 9. compute ratio2 = sum(degree(all neighbors of $v_{j}$))/sum(second_neighbor_degree($v_{j}$)) 10.$\;Ts_{2}$ = (pow(ratio2,$\;q_{i}$) − ratio2)/(1-$q_{i}$) 11. **End For** 12. compute $IC\left(v_{i}\right)=Ts_{1}\left(v_{i}\right)+\alpha_{i}Ts_{2}\left(v_{i}\right)\;$ 13. **For** node $v_{j}$ in $N_{i}$ do 14. $SI\left(v_{i}\right)\;$= sum($IC\left(v_{j}\right)$) 15. **End For** 16. **For** node $v_{j}$ in $N_{i}$ do 17. $\;TRank\left(v_{i}\right)\;$= sum(SI$\left(v_{j}\right)$) 18. **End For**


In this algorithm, lines 3 to 11 are the core, and lines 3 to 6, respectively, calculate $Ts_{1}$ for each node. The time complexity is O(n∗k). n represents the number of nodes, and k is related to the number of neighbors. The $Ts_{2}$ of each node is calculated in lines 7 to 11. The time complexity is O(n∗k∗m), where m is related to the number of secondary neighbors of the node. Therefore, the overall complexity of the algorithm is O(n∗k∗m).

## 4. Experiment

In this section, we will evaluate the comprehensive ability of TRank from three aspects: identification, correctness, and efficiency. At the same time, we will use the infectious disease model to simulate the process of information transmission in the real network, so as to better evaluate the transmission ability of nodes.

### 4.1. Network Datasets

In this paper, six random synthetic Barabasi Albert (BA) scale-free networks [28] of different sizes, four random synthetic Fractional Preferential Attachment (FPA) scale-free networks [29] of different ‘f’ parameter, and 10 real networks of different sizes are selected. Table 1 shows the analysis data of 10 random synthetic scale-free networks, and Table 2 shows the analysis data of 10 real networks, including the number of nodes, the number of edges, the average degree, the maximum degree, the assortativity, and the clustering coefficient.

Some of these network datasets are detailed below.

(1) BA network is a scale-free network and whose degree distribution follows a power law, it certainly contains few nodes with unusually high degree as compared to the other nodes of the network. We set the number of nodes and average degree of BA model to synthesize six random networks of different sizes.

(2) FPA network is a generalization of BA network. When compared with the BA network, FPA network is acyclic. The element controlling the FPA model properties is the ‘f’ parameter (where f ∈ (0,1). For f = 1, FPA model implements the classical BA model). We set the f parameter of FPA model to synthesize four networks of the same size.

(3) The Karate network has 34 members of a karate club. After more than two years of continuous time, Zachary calculated 78 sides to represent their relationships according to the level of interpersonal communication. Because of the conflict between the instructor and the manager at some time, their relationships broke down, resulting in two factions.

(4) The Dolphin data set has 62 nodes, representing dolphins from two families. It took more than seven years of continuous observation to form the data set. Lusseau et al. counted the degree of interaction between each pair of dolphins and used 159 edges to describe the relationships between them.

(5) The Jazz dataset has 198 nodes, each of which is a jazz musician, and the edges represent two musicians playing together in a band.

(6) Elegans represents the metabolic network of Caenorhabditis elegans. The metabolic network is composed of nodes and substrates. These nodes and substrates are connected by links, which are the actual metabolic responses.

(7) The Email dataset represents the email communication network of Rovira I Virgili University in Taragona, southern Catalonia, Spain. Each node is a user, and each edge indicates that at least one email has been sent. 

(8) Euroroad is the international electronic road network, which is mainly located in Europe. The network is undirected. Each node represents a city, and the edge between two nodes represents that they are connected by an E-road.

(9) The East data set describes the interaction network composed of proteins, which can be used to discover the interaction among thousands of proteins. It is very important for biology to recognize the correlation of large-scale data sets.

(10) The Hamsterster network contains the friendships and family links between the users of the website.

(11) Powergrid is an undirected network that contains information about the western power grid of the United States of America. The connection between two points represents a power line, and a node can be a generator, transformer or substation.

(12) PGP is the pretty good privacy (PGP) algorithm user interaction network.

### 4.2. TsallisRank Algorithm Recognition Analysis

This paper will use the degree centrality (DC) [4], K-shell (KS) [15], local entropy (LE) [30], mixed degree decision (MDD) [16], and extended neighborhood core centrality (Cnc+) [17] as the comparison metrics in order to better evaluate the rank algorithm.

This part of the experiment mainly verifies the ability of the algorithm to identify the influential nodes in the network, among which the verification methods are the D method, the CCDF method, and the M method.

- D method

(13) $$D=\frac{number\;of\;distinct\;ranks\;}{n}$$

where n represents the number of network nodes. The maximum value of function D is 1. It means that, in the network, each node has a unique influence ability, and each node can be effectively distinguished. The minimum value of function D is 1/n, which means that all the nodes have the same influence ability. At this time, the recognition ability of the algorithm is the worst. In this paper, the D method is applied to each algorithm, which can effectively distinguish the recognition ability of each method.

- CCDF method

(14) $$\mathrm{CCDF}\left(r\right)=\frac{n-\sum_{i=1}^{r}n_{i}}{n}$$

where n represents the total number of network nodes and $n_{i}$ represents the number of nodes occupied by rank variable r in a ranking list. With the increase of rank r, the functional value falls faster, and the ranking distribution performance worsens. The CCDF (comprehensive cumulative distribution function) method seeks the ranking distribution of different methods, and the ranking variable r determines the value of the function.

- M method

(15) $$M\left(R\right)={\left(1-\frac{\sum_{r\in R}n_{r}\left(n_{r}-1\right)}{n\left(n-1\right)}\right)}^{2}$$

where n is the number of different rankings in the R ranking list, and $n_{r}$ is the number of nodes occupying the same ranking R. If all nodes have the same ranking, the value of M is 0. If all nodes have different rankings, the closer the value of function M is to 1, the better the recognition of this ranking list.

- Jaccard similarity coefficient

(16) $$J_{c}\left(X,Y\right)=\frac{\left|X\left(c\right)\cap^{\;}Y\left(c\right)\right|}{\left|X\left(c\right)\cup^{\;}Y\left(c\right)\right|}$$

where the Jaccard similarity coefficient is used to determine the degree of similarity of two rankings. In list X, X(c) represents the set of the first c rankings. The closer the value of $J_{c}$ is to 1, the more similar the two rankings are. In addition, it also verifies the high accuracy of ranking R.

**Experiment 1:**

**Figure 3:**
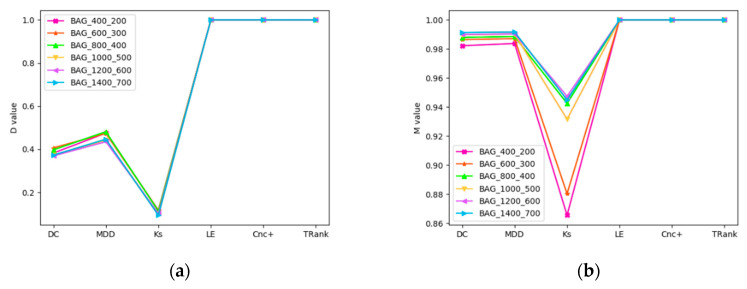

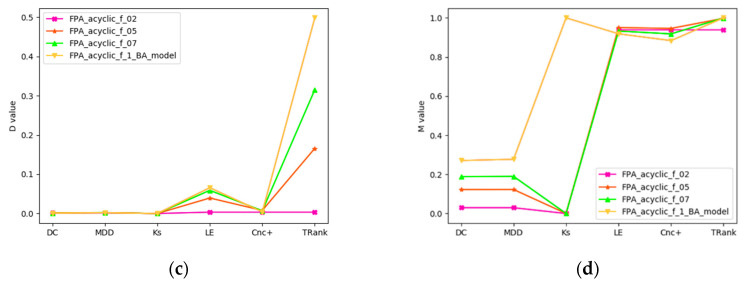
D and M curves of ranking methods in random synthetic scale-free networks: (**a**) D curve in BA networks; (**b**) M curve in BA networks; (**c**) D curve in FPA networks; (**d**) M curve in FPA networks.

Experiment 1 is mainly to verify the recognition ability of the algorithm in random synthetic scale-free networks with D method and M method. We can see that some central methods do not perform well, as shown in Figure 3. For example, DC, MDD and KS have lower M and D values in all networks. In BA networks, Le, Cnc+ and TRank perform best. KS performance is the worst, and as the number of nodes in BA network decreases, the performance gets worse. In FPA networks, TRank performs best. Although the M values of Le and Cnc+ are very high, the D values are low.

**Experiment 2:**

Experiment 2 will use the D method to verify the recognition ability of the algorithm in real networks. D (x) shows the functional D value of method X for different datasets, as shown in Table 3. DC, MDD, and KS do not perform well, similar to experiment 1. In addition, Cnc+ is highly recognizable in some networks, and LE only performs better than TRank in the karate network.

**Experiment 3:**

**Figure 4:**
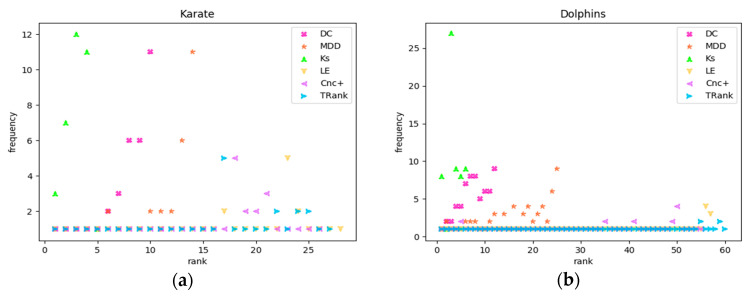

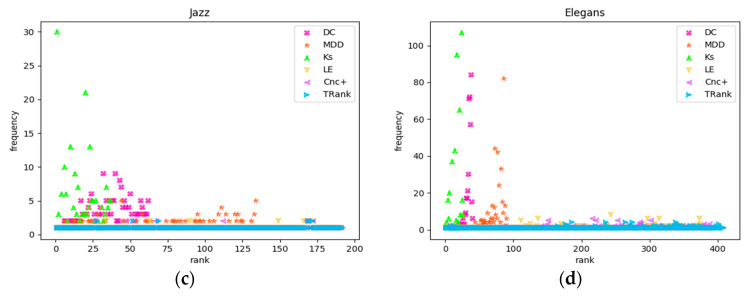
Node rankings and frequency distributions of the ranking methods: (**a**) In Karate network; (**b**) In Dolphins network; (**c**) In Jazz network; (**d**) In Elegans network.

Experiment 3 takes ranking as the abscissa and the number of nodes in each ranking as the frequency so that the degree of recognition of different methods can be more clearly seen in order to view the frequencies of the nodes in each ranking. The closer the frequency of nodes is to 1, the better the recognition ability of the ranking method. As shown in Figure 4, in the four real networks of Karate, Dolphin, Jazz, and Elegans, the frequencies of the ranking nodes of DC, KS, and MDD are scattered above the frequency of 1 while those of LE and TRank is always around the frequency of 1.

**Experiment 4:**

**Figure 5:**
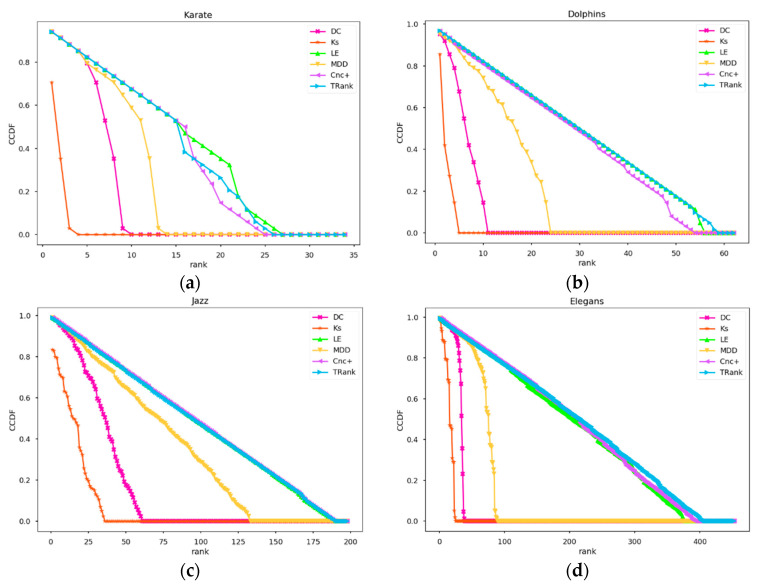
CCDF curves of the ranking methods: (**a**) In Karate network; (**b**) In Dolphins network; (**c**) In Jazz network; (**d**) In Elegans network.

Experiment 4 will explore the ranking distributions of different methods. It uses the CCDF to draw the distributions of the four networks, including Karate, Dolphin, Jazz, and Elegans, using different algorithms, as shown in Figure 5. From Figure 5, we can see that DC, KS, and MDD fall rapidly in the four networks. In the small Karate network, the performance is very good. Cnc+ is very close to the TRank, but the TRank still decreases at a slower rate.

**Experiment 5:**

Table 4 shows the ranking lists of the M method applied to different methods for the 10 real networks. In this table, M(x) shows the M values of the function for different datasets. As can be seen from Table 4, LE, Cnc+, and bank all have extremely high scores. DC, KS, and MDD perform poorly on multiple networks.

### 4.3. Algorithm Correctness

This paper uses the SIR [31] model to obtain the propagation impacts of network nodes in order to verify the correctness of the ranking method. In the simulation process [32], at the beginning of this paper, node v is initialized as the infected state, and other nodes are set to vulnerable states. In each iteration, the infected node tries to infect all the neighboring nodes in its vulnerable state with probability *β*. Subsequently, it changes to the recovered state by itself, and repeats this process until no node in the network is in the infected state. At the end of the infection process, the number of recovered nodes is regarded as the propagation ability of node v. In the infectious disease model, *β* is set to float near the infection threshold $\beta_{\mathrm{th}}\;\sim \;\langle k\rangle /\langle k^{2}\rangle$, where $\langle k\rangle /\langle k^{2}\rangle$ represents the average degrees of the first level neighbor and the second level neighbor, respectively. Because of the randomness of the iterative process of the disease model, this paper decided to simulate the process repeatedly for each node and then take the average value. The simulations will follow the following rules: for networks |V|<100, the simulation is iterated $10^{4}$ times; for 100<|V|<$10^{4}$, the simulation is iterated $10^{3}$ times; and, for |V|>$10^{4}$, the simulation is iterated 100 times.

At the end of the SIR simulations, the σ ranking is obtained, and the correlation coefficient of Kendall’s tau [33] is compared with the R ranking calculated by each algorithm. In order to quantify the correctness of the different methods, it is assumed that $\left(x_{1},y_{1}\right),\left(x_{2},y_{2}\right),\ldots ,\left(x_{n},y_{n}\right)$ is a group of rankings of lists X and Y, respectively. For any pair of $\left(x_{i},y_{i}\right)$ and $\left(x_{j},y_{j}\right)$, if ($x_{i}>x_{j}$ and $y_{i}>y_{j}$) or ($x_{i}<x_{j}$ and $y_{i}<y_{j}$) is satisfied, it shows that they are consistent. If ($x_{i}<x_{j}$ and $y_{i}>y_{j}$) or ($x_{i}>x_{j}$ and $y_{i}<y_{j}$), it demonstrates that they are inconsistent. If $x_{i}=x_{j}$ and $y_{i}=y_{j}$, they are both inconsistent. When considering these relations, Kendall’s tau τ rank correlation coefficient is defined as Equation (15).$\;n_{c}$ and $n_{d}$ represent the numbers of agreements (c) and disagreements (d), respectively. n is the size of the rank list.

(17) $$\tau \left(R,\sigma \right)=\frac{n_{c}-n_{d}}{0.5n\left(n-1\right)}$$

**Experiment 6:**

Experiment 6 will calculate the correlation coefficient τ between the ranking lists of different methods and the σ ranking obtained in the infectious disease model. Table 5 presents the specific results. In this table, $\beta_{\mathrm{th}}$ is the threshold *β* of the actual infection probability. τ(x,σ) column shows Kendall’s tau correlation coefficient of methods x and σ. It can be seen from the table that compared with other methods, the rank r calculated by TRank is extremely correlated with σ. Only in the karate and PGP networks, where Cnc+ exceeds TRank, is it the most correlated with σ. DC, KS, and LE have low correlations with σ.

**Experiment 7:**

**Figure 6:**
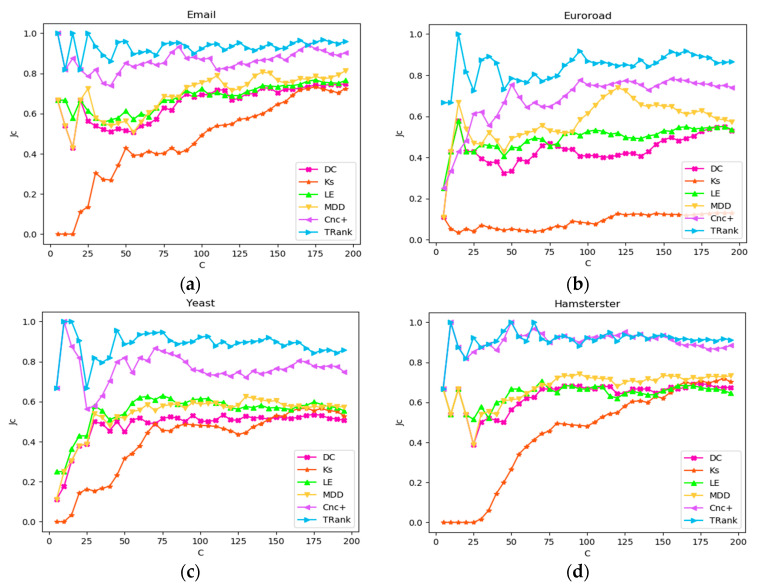
Ranking lists and the curves of the Jaccard similarity coefficient of σ: (**a**) In Email network; (**b**) In Euroroad network; (**c**) In Yeast network; (**d**) In Hamsterster network.

In this experiment, the Jaccard similarity coefficient will be used to determine the degree of similarity of two rankings. In list x, X(c) represents the set of the first C rankings. The smaller that $J_{c}$ is, the more similar the two rankings. in addition, the higher accuracy of ranking R is verified. Figure 6 shows the four networks of Email, Euroroad, Yeast, and Hamsterster. For networks with less than 200 network nodes, the maximum value of ranking variable C is the number of network nodes. For networks with more than 200 network nodes, the maximum value of ranking variable C is 200. From the experimental results, TRank has good performance in the four networks; Cnc+ only has similar performance with TRank in the Hamsterster network; and the rest of the algorithms, such as DC, KS, Le, and MDD, show slow upward trends at the beginning, and finally remain stable at the bottom of the TRank curve.

**Experiment 8:**

**Figure 7:**
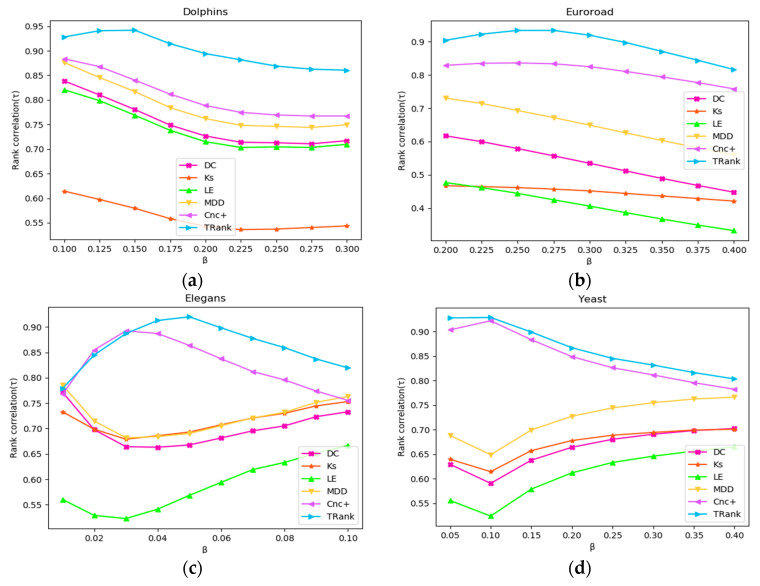
Relationships between SIR *β* and Kendall’s tau of the ranking lists: (**a**) In Dolphins network; (**b**) In Euroroad network; (**c**) In Elegans network; (**d**) In Yeast network.

In this paper, *β* is used as a variable to carry out the SIR simulation in the Karate, Euroroad, Elegans, and Yeast networks, and different σ lists are obtained. The Kendall’s tau correlation coefficient rankings of each method and different σ lists are calculated. The results of the experiment are shown in Figure 7. According to Figure 7, in the Dolphin and Euroroad networks, as *β* increases, the correlation between various algorithms and σ shows a downward trend. However, in the Elegans and Yeast networks, the curves of LE, DC, MDD, and KS first decline and then rise, and only the curves of Cnc+ and TRank first rise and then fall.

**Experiment 9:**

**Figure 8:**
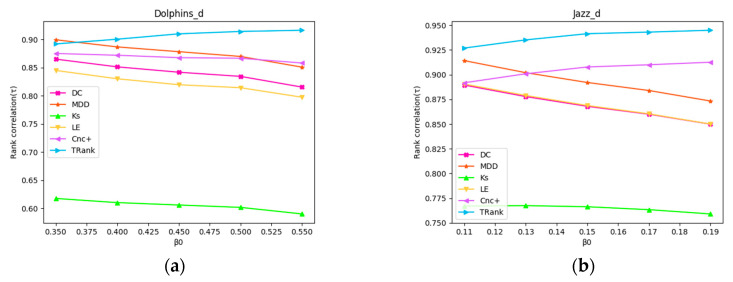
Relationships between modified SIR $\beta_{0}$ and Kendall’s tau of the ranking lists: (**a**) In Dolphins network; (**b**) In Jazz network.

In order to simulate the infection process more realistically, we modify the SIR model by adding a natural decay function: $\beta_{t}=\beta_{0}e^{-t}$, where $\beta_{0}$ is the initial value of infection probability, t is the step of iterations. The infection probability of each iteration decreases gradually. Modified SIR simulation is carried out in Dolphins and Jazz network, and the process are the same as Experiment 8. The results of the experiment are shown in Figure 8. With the increase of $\beta_{0}$, the correlation of Le, DC, MDD, and KS in the two networks show a downward trend, while Cnc+ gradually decreases in Dolphins network and increases gradually in Jazz network. The correlation of TRank is the highest and increases gradually in both networks.

### 4.4. Algorithm Efficiency

**Experiment 10:**

**Figure 9:**
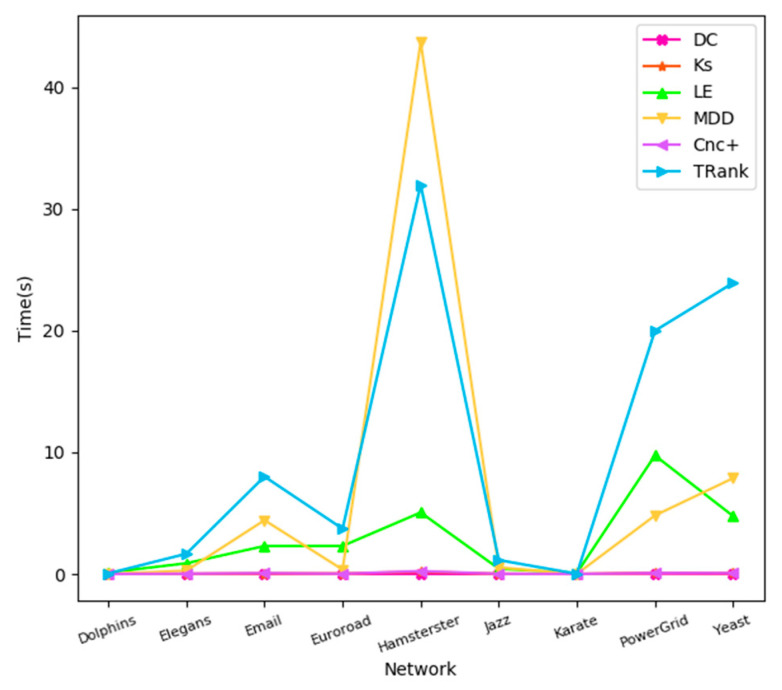
Time curves of different algorithms in real networks.

In this part, we will look at the time consumed by each algorithm in different networks. The experimental environments are as follows: python = 3.6, numpy = 1.16, and pandas = 0.24. In nine real networks, the time consumption of the 6 algorithms are quite different, among which the DC, KS, and Cnc+ based on degrees are relatively simple, and so their time consumptions are very small and remain stable, as shown in Figure 9. However, slightly complex algorithms, such as MDD, LE, and TRank, will take longer.

## 5. Discussion and Conclusions

In this paper, we propose an effective ranking algorithm TsallisRank, which solves the problem that the traditional method lacks the ability to capture the global information of nodes. In addition, this method considers the positions of nodes in the network. In this method, we consider the influence of the numbers of primary neighbors and secondary neighbors on a node’s propagation ability. Furthermore, we use Tsallis in order to evaluate the characteristics of the network structure, which can better evaluate the influential nodes in the network. By simulating the SIR infection process using real networks, the diffusion ability of each node in the network is obtained, and then the ranking list of the ranking methods is obtained. Kendall’s tau correlation coefficient analysis is carried out, and it is found that TRank can effectively rank the affected nodes; when compared with other methods, such as DC, KS, MDD, Cnc, and LE, TRank is more accurate and effective. However, in terms of time consumption, the performance of TRank is not outstanding, so it needs to be optimized in a follow-up work. Compared with DC, the TRank algorithm is more complex, which leads to a great increase in the computing time, which is also a limitation to the algorithm. 

## Figures and Tables

**Table 1:** Some statistical data of random synthetic scale-free networks.

Network	|V|	|E|	Average Degree	Maximum Degree	Assortativity	Clustering Coefficient
BAG_400_200	400	40,000	200.0	384	−0.398111	0.722654
BAG_600_300	600	90,000	300.0	577	−0.397054	0.721492
BAG_800_400	800	160,000	400.0	776	−0.397650	0.724718
BAG_1000_500	1000	250,000	500.0	967	−0.397126	0.722987
BAG_1200_600	1200	360,000	600.0	1159	−0.397411	0.723613
BAG_1400_700	1400	490,000	700.0	1347	−0.397143	0.723255
FPA_acyclic_f_1_BA_model	100,006	100,005	1.99998	1340	−0.014383	0.0
FPA_acyclic_f_07	100,006	100,005	1.99998	1621	−0.028993	0.0
FPA_acyclic_f_05	100,006	100,005	1.99998	4981	−0.047784	0.0
FPA_acyclic_f_02	100,006	100,005	1.99998	21,951	−0.157886	0.0

**Table 2:** Some statistical data of real networks.

Network	|V|	|E|	Average Degree	Maximum Degree	Assortativity	Clustering Coefficient
Karate	34	78	4.588	17	−0.4756	0.5706
Dolphins	62	159	5.129	12	−0.043594	0.2590
Jazz	198	2742	27.697	100	0.0202	0.6175
Elegans	453	2025	8.940	237	−0.2258	0.6465
Email	1133	5451	9.622	71	0.0782	0.2203
Euroroad	1174	1417	2.414	10	0.1267	0.0167
Yeast	2361	7182	6.0839	66	−0.0846	0.1301
Hamsterster	2426	16,631	13.711	273	0.0474	0.5376
PowerGrid	4941	6594	2.669	273	0.0035	0.0801
PGP	10,680	24,316	4.554	205	0.2382	0.2659

**Table 3:** D method evaluation performance analysis table.

Network	D(DC)	D(Ks)	D(LE)	D(MDD)	D(Cnc+)	D(TRank)
Karate	0.3235	0.1471	**0.8235**	0.4412	0.7647	**0.7941**
Dolphins	0.1935	0.0968	0.9194	0.4032	0.8871	**0.9677**
Jazz	0.3131	0.1869	0.9646	0.6768	0.9646	**0.9697**
Elegans	0.0883	0.0574	0.8366	0.1987	0.8676	**0.9029**
Email	0.0424	0.0477	0.8914	0.1703	0.9170	**0.9762**
Euroroad	0.0077	0.0068	0.1806	0.0187	0.0707	**0.9446**
Yeast	0.0237	0.0216	0.6357	0.0923	0.6192	**0.7954**
Hamsterster	0.0458	0.0528	0.6587	0.1620	0.6686	**0.7003**
PowerGrid	0.0032	0.0040	0.2117	0.0105	0.0565	**0.9041**
PGP	0.0078	0.0124	0.3727	0.0329	0.2902	**0.7456**

**Table 4:** M method analysis table.

Network	M(DC)	M(Ks)	M(LE)	M(MDD)	M(Cnc+)	M(TRank)
Karate	0.7079	0.5499	0.9577	0.7536	0.9472	**0.9542**
Dolphins	0.8312	0.5576	0.9905	0.9091	0.9895	**0.9979**
Jazz	0.9659	0.8951	0.9993	0.9911	0.9993	**0.9994**
Elegans	0.7922	0.7399	0.9972	0.8768	0.9980	**0.9988**
Email	0.8874	0.8521	0.9990	0.9233	0.9997	**0.9999**
Euroroad	0.4442	0.3312	0.9181	0.6510	0.9463	**0.9990**
Yeast	0.7472	0.7052	0.9921	0.7477	0.9962	**0.9972**
Hamsterster	0.8980	0.8907	0.9853	0.9274	0.9856	**0.9858**
PowerGrid	0.5927	0.3713	0.9635	0.6940	0.9568	**0.9999**
PGP	0.6193	0.5000	0.9781	0.6679	0.9939	**0.9997**

**Table 5:** Correlation coefficients of SIR and Kendall.

Network	β	$\beta_{th}$	τ(σ,DC)	τ(σ,Ks)	τ(σ,LE)	τ(σ,MDD)	τ(σ,Cnc+)	τ(σ, TRank)
Karate	0.250	0.129	0.6310	0.5490	0.6542	0.6542	**0.9269**	0.8128
Dolphins	0.150	0.147	0.7805	0.5796	0.7689	0.8170	0.8403	**0.9418**
Jazz	0.040	0.026	0.8371	0.7847	0.8415	0.8663	0.9455	**0.9726**
Elegans	0.050	0.025	0.6677	0.6931	0.5685	0.6902	0.8636	**0.9199**
Email	0.050	0.054	0.7892	0.7962	0.7654	0.8073	0.9413	**0.9578**
Euroroad	0.275	0.333	0.5572	0.4571	0.4249	0.6721	0.8337	**0.9341**
Yeast	0.100	0.061	0.5908	0.6147	0.5241	0.6490	0.9222	**0.9289**
Hamsterster	0.020	0.024	0.7447	0.7333	0.6416	0.7510	0.9234	**0.9349**
PowerGrid	0.200	0.258	0.6244	0.4503	0.5055	0.6667	0.7887	**0.9107**
PGP	0.100	0.053	0.3644	0.3651	0.2026	0.3745	**0.7840**	0.6913
